# A new species of leech of the genus *Placobdella* (Hirudinida, Glossiphoniidae) from the American alligator (*Alligator mississippiensis*) in Mississippi, USA

**DOI:** 10.3897/zookeys.667.10680

**Published:** 2017-04-10

**Authors:** Dennis J. Richardson, William E. Moser, Charlotte I. Hammond, Eric A. Lazo-Wasem, Chris T. McAllister, Eric E. Pulis

**Affiliations:** 1 Department of Biological Sciences, Quinnipiac University, 275 Mt. Carmel Avenue, Hamden, Connecticut, 06518 USA; 2 Smithsonian Institution, National Museum of Natural History, Department of Invertebrate Zoology, Museum Support Center MRC 534, 4210 Silver Hill Road, Suitland, Maryland, 20746 USA; 3 Division of Invertebrate Zoology, Peabody Museum of Natural History, Yale University, P.O. Box 208118, New Haven, Connecticut, 06520 USA; 4 Science and Mathematics Division, Eastern Oklahoma State University, 2805 NE Lincoln Road, Idabel, Oklahoma, 74745 USA; 5 Institute for Marine Mammal Studies, 10801 Dolphin Lane, Gulfport, Mississippi, 39503 USA

**Keywords:** *Placobdella siddalli*, *Alligator mississippiensis*, American Alligator, Glossiphoniidae, Hirudinea, Clitellata

## Abstract

To date, the only species of leech reported from the American Alligator, *Alligator mississippiensis* is *Placobdella multilineata*. Seven specimens of a previously undescribed species of *Placobdella* were collected from the feet and lower jaw of a single female alligator from the Pascagoula River Wildlife Management Area, George County, Mississippi. The new species was named *Placobdella siddalli* Richardson & Moser, **sp. n.**, in honor of the contributions of Dr. Mark Siddall to our understanding of the biology of leeches. *Placobdella siddalli* Richardson & Moser is similar to other papillated members of the genus *Placobdella*, but differs from *Placobdella ali* Hughes & Siddall, 2007, *Placobdella rugosa* (Verrill, 1874), *Placobdella multilineata* Moore, 1953, and *Placobdella papillifera* (Verrill, 1872) in coloration, papillation, ventral striping, and in the possession of a relatively large caudal sucker. In addition, molecular comparison of 626 nucleotides of CO-I between the new species and other papillated leeches (*P. ali*, *P. multilineata*, *Placobdella ornata*, *P. papillifera*, *P. rugosa*) revealed interspecific differences of 14.0–18.0% (88–113 nucleotides).

## Introduction

There are 22 recognized species of Glossiphoniid leeches in the genus *Placobdella* parasitizing birds, mammals, amphibians and reptiles (Moser et al. 2014). To date, the only species of leech reported from the American Alligator, *Alligator mississippiensis* (Daudin, 1802) Cuvier, 1807 is *Placobdella multilineata* Moore, 1953. *Placobdella multilineata* is a generalist parasite of reptiles having been reported from turtles, snakes, and alligators from throughout the southeastern United States and Mississippi River Valley as far north as Illinois and Iowa (Klemm 1985; Moser et al. 2014). In addition, Saumure and Doody (1998) reported two specimens of *P. multilineata* from a three-toe Amphiuma, *Amphiuma tridactylum* Cuvier, 1827 from Louisiana. In the course of a routine parasitological survey of blood parasites of Mississippi alligators, seven specimens of a previously undescribed species of *Placobdella* were collected and are described herein.

## Materials and methods

On 9 August 2015, seven specimens of a previously undescribed species of *Placobdella* were collected from the feet and lower jaw of a single female Mississippi alligator, approximately 1.2 m long, that was pole snared from Davis Eddy, a cypress swamp constituting an oxbow lake of the Pascagoula River in the Pascagoula River Wildlife Management Area, George County, Mississippi (30°54'11"N, 088°44'35"W). Six additional alligators examined from the same region were leech-free.

Leeches were relaxed, fixed and examined as described by Moser et al. (2006). Terminology for plane shapes follows Clopton (2004). Ranges are given followed by mean in parentheses. One specimen was mounted in Canada balsam following the techniques of Richardson and Barger (2006). Specimens were deposited in the Invertebrate Zoology Collection of the Peabody Museum of Natural History, Yale University, New Haven, Connecticut, USA (YPM) and the Smithsonian Institution, National Museum of Natural History, Washington, District of Columbia, USA (USNM). The following specimens held in the collection of the Peabody Museum of Natural History, Yale University, New Haven Connecticut were examined in comparison to *Placobdella siddalli* Richardson & Moser, and caudal sucker diameter relative to body width and length was determined: *Placobdella ali* Hughes & Siddall, 2007 (YPM 047497, N = 1; YPM 058254, N = 1; and YPM 058279, N = 1), *P. multilineata* (YPM 083513, N = 8), *Placobdella ornata* (Verrill, 1827) Moore, 1901 (YPM 048007, N = 1; YPM 058351, N = 3, YPM 058360, N = 1; YPM 058371, N = 1; YPM 058551, N = 1; and YPM 058322, N = 2), *Placobdella papillifera* (Verrill, 1872) Moore, 1952 (YPM 043493, N = 1; YPM 043494, N = 7; and YPM 043557, N = 3), *Placobdella parasitica* (Say, 1824) Moore 1901 (YPM 053088, N = 1; YPM 058096, N = 1; YPM 058091, N = 1; YPM 058092, N = 1; YPM 058093, N = 1; and YPM 058094, N = 1), and *Placobdella rugosa* (Verrill, 1874) Moore, 1901 (YPM 056679, N = 1; YPM 056680, N = 2; YPM 056681, N = 1; and YPM 058081, N = 2).

Molecular analyses were conducted according to Richardson et al. (2010) as follows: For the proteinase K treatment step, tissue samples were taken from the caudal suckers of individual leeches and lysed overnight at 56^o^C. DNA was isolated with the DNeasy Blood & Tissue Kit from Qiagen (Cat. No. 69504), following the protocol given for the purification of total DNA from animal tissues (spin-column). DNA was eluted from the spin columns with 100 µl of buffer.

Polymerase Chain Reactions (PCR) were prepared using the Illustra PuRe Taq Ready-To-Go PCR beads from GE Health Care (Cat. No. 27-9559-01). Primers were purchased from Invitrogen and were comprised of two primers each for mitochondrial cytochrome c oxidase subunit I (CO-I) as specified by Light and Siddall (1999). Specifically, the CO-I primers were LCO1490 (5’GGTCAACAAATCATAAAGATATTGG 3’) and HCO2198 (5’TAAACTTCAGGGTGACCAAAAAATCA 3’). Final volume of PCR reactions was 25 µl with three µl of leech genomic DNA added per reaction. DNA was amplified under the following PCR conditions: 94^o^C for five min.; 35 cycles of (94^o^C for 30 sec, 50^o^C for 30 sec, 72^o^C for 45 sec); 72^o^C for seven min. Following PCR, samples were cleaned up using a QIAquick PCR purification kit from Qiagen (Cat. No. 28104).

Purified PCR products were sequenced using the HCO2198 primer for the cytochrome c oxidase subunit I gene by the W. M. Keck Foundation Biotechnology Resource Laboratory at Yale University. The DNA sequences were aligned using Clustal W version 2 (Larkin et al. 2007) and checked manually using SeaView 4 (Gouy et al. 2010) and then analyzed using PAUP* 4.0b10 (Swofford 2002) and compared to other leech DNA sequences contained within Genbank. Uncorrected p distances were calculated using PAUP*.

## Results

### Species description

#### Placobdella siddalli

Taxon classificationAnimaliaRhynchobdellidaGlossiphoniidae

Richardson & Moser sp. n.

http://zoobank.org/9170DAD3-1657-4000-BF99-474E6DCDCB91

**Figure 1.**
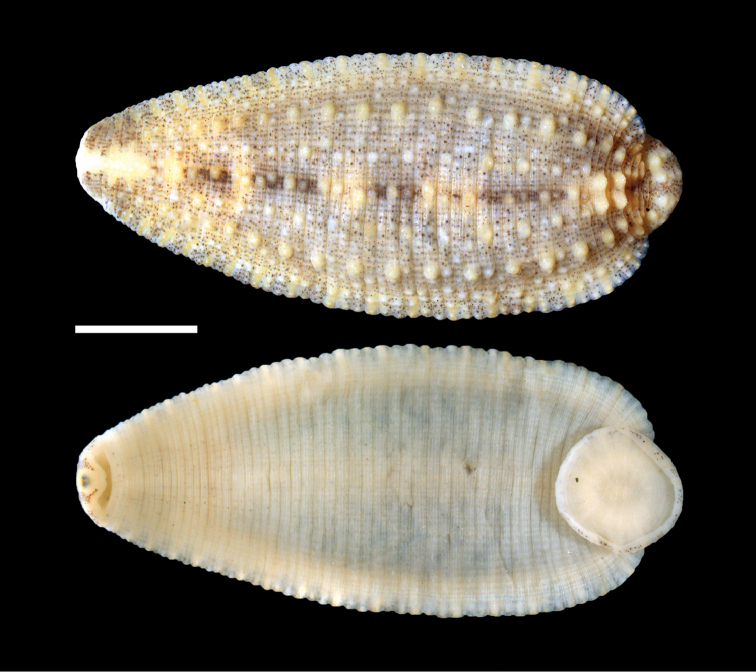
Holotype specimen of *Placobdella siddalli* Richardson & Moser, YPM 083857 **A** Dorsal surface **B** Ventral surface. Scale bar: 2 mm.

**Figure 2.**
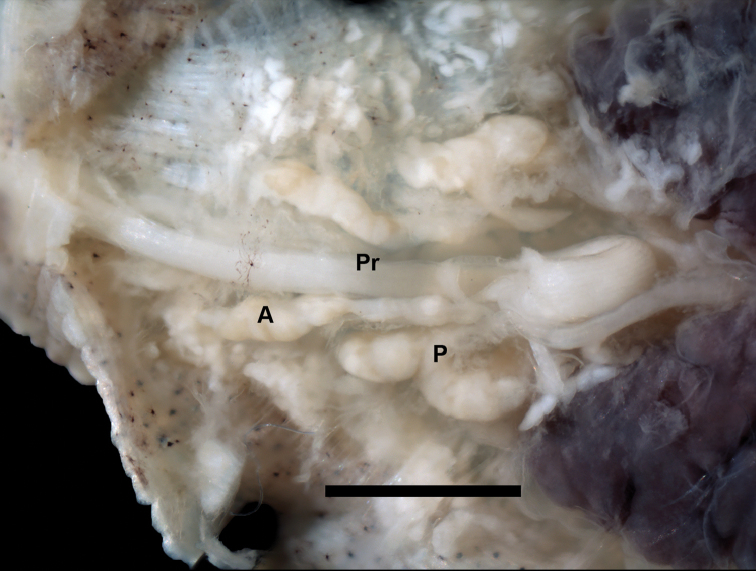
Internal anatomy of *Placobdella siddalli* Richardson & Moser, YPM 083875. Dorsal view, anterior salivary gland (**A**), posterior salivary gland (**P**), proboscis (**Pr**). Scale bar: 1 mm.

**Figure 3.**
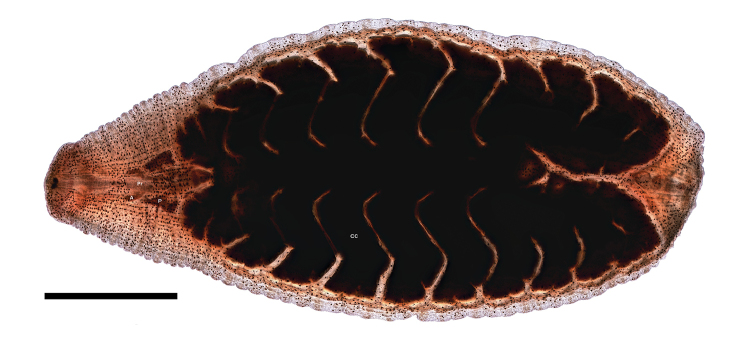
Paratype specimen of *Placobdella siddalli* Richardson & Moser, YPM 083876 mounted in Canada balsam, crop ceca (**CC**), anterior salivary gland (**A**), posterior salivary gland (**P**), proboscis (**Pr**). Scale bar: 2 mm.

Figures 1 , 2 , 3 ; 5 , 6

##### Material examined.

Holotype (YPM 083857) Davis Eddy, a cypress swam constituting an oxbow lake of the Pascagoula River in the Pascagoula River Wildlife Management Area, George County, Mississippi (30°54'11"N, 088°44'35"W).

Paratypes (YPM 083875-083876, YPM 090164-090165; USNM 1422202-1422203) Davis Eddy, a cypress swam constituting an oxbow lake of the Pascagoula River in the Pascagoula River Wildlife Management Area, George County, Mississippi (30°54'11"N, 088°44'35"W).

##### Morphological description.

*External morphology*: (Fig. 1) Body elliptoid; length of preserved specimens 9.0–11.1 (9.8) mm, width at widest point (in center of body) 3.6–5.0 (4.5) mm. Dorsum base color beige with olive-green pigment spots. Dorsal papillae arranged in five rows (dorsomedial, two paramedial and two paralateral rows of unpigmented, stellate papillae) with repeating patterns of papillae size as follows: dorsomedial papillus of neural annulus large; paramedial papillae of neural annulus small; paralateral papillae of neural annulus large. Dorsomedial papillus of annulus posterior to neural annulus small; paramedial papillae of annulus posterior to neural annulus large; paralaterial papillae of annulus posterior to neural annulus small. Dorsomedial papillus of annulus anterior to neural annulus greatly reduced (sensillus); paramedial and paralateral papillae lacking on annulus anterior to neural annulus. Lateral papillae much less organized, not in distinct rows. Lateral region with alternating unpigmented and modestly pigmented sections (being characterized by small chromatophores). Anal opening located in furrow, one anteriad annulus from the caudal sucker. Beginning adjacent to the anus and commencing anteriad are two rows of three papillae, followed by two pairs of prominent paramedial papillae. Two pair of near-coalesced eyespots, typical of the genus *Placobdella*, within lateral unpigmented mask that extends posteriad into interrupted dorsal-medial pigment line that extends posteriorly to anus. Most pronounced pigmentation of dorsal-medial pigment line from genital region to anterior pair of prominent paramedial papillae. Caudal sucker orbicular, diameter 2.0–2.2 (2.1) mm; 18.64–22.9 (21.1) % of the length leech; dorsal surface with approximately three rows of papillae, the anterior-most of which is most prominent. Ventral surface of the whole body with scattered chromatophores, most concentrated in genital region and without stripes.

*Internal morphology*: (Figs 2 and 3) Proboscis pore on rim/lip of anterior sucker. Blunt-tipped muscular proboscis nearly uniformly cylindrical, only very modest enlargement at base. Two pair of discrete salivary glands. Anterior pair very narrowly doliform to oblong and slightly enlarged anteriad; ductal medially inserted into narrowly elliotoid posterior salivary glands; ductal of anterior salivary gland anastomoses with ductual of posterior salivary gland half-way between the posterior salivary gland and proboscis forming common duct. Esophagus extends from the base of the proboscis with one pair sac-like mycetomes. Seven pairs of diverticulated crop ceca, last pair extending posteriorly and diverticulated into four sections. Four pairs of intestinal ceca.

*Reproductive system*: Male and female gonopores in furrows and separated by two annuli. Six pair of testisacs.

##### Taxonomic summary.

Type host. American Alligator, *Alligator mississippiensis* (Daudin, 1802) Cuvier, 1807

##### Type locality.

Davis Eddy, a cypress swamp constituting an oxbow lake of the Pascagoula River in the Pascagoula River Wildlife Management Area, George County, Mississippi (30°54'11"N, 088°44'35"W).

##### Type material.

YPM 083857 (Holotype), YPM 083875-083876, YPM 090164-090165 (Paratypes), USNM 1422202-1422203 (Paratypes).

##### Etymology.

The specific epithet *siddalli* is in honor of Dr. Mark Siddall in recognition of the profound advancements that he has contributed to our understanding of glossiphoniid leeches, particularly in regard to the taxonomic importance of preanal papillae.

##### Molecular description.

Molecular characterization is based on sequence of 626 nucleotides of the mitochondrial Cytochrome c oxidase subunit I (GenBank KY780962). Molecular comparison of 626 nucleotides of CO-I revealed 100% identify between two specimens of *Placobdella siddalli* Richardson & Moser collected from the same host in Davis Eddy, George County, Mississippi (type locality; YPM 083876, GenBank KY780962 and an interspecific difference of 14.0% to 15.7% (88 to 98 nucleotides) between *P. siddalli* Richardson & Moser and four specimens of *P. multilineata* from Louisiana, Mississippi, and Oklahoma. Additional intraspecific differences of 15.9% to 16.7% (99 to 105 nucleotides) were found between *P. siddalli* Richardson & Moser and four specimens of *P. rugosa* collected from the type locality (North Dakota; GenBank JX412986-JX412990); difference of 18.0% (113 nucleotides) between *P. siddalli* Richardson & Moser and three specimens of *P. ali* from the type locality (New York) and Connecticut (GenBank HM347040–HM347042); differences of 16.9% to 17.3% (106 to 109 nucleotides) between *P. siddalli* Richardson & Moser and five specimens of *P. papillifera* (GenBank KC505241–KC505245) from its type locality (West River, New Haven, New Haven County, Connecticut); differences of 15.0% to 15.3% (94 to 96 nucleotides) between *P. siddalli* Richardson & Moser and five specimens of *P. ornata* (GenBank JQ812128–JQ812132) collected from the type locality (West River, New Haven County, Connecticut); and differences of 14.7% and 14.8% (92 to 93 nucleotides) between *P. siddalli* Richardson & Moser and five specimens of *P. parasitica* collected from the type locality (Minnesota; GenBank KF058895–KF058899).

**Figure 4.**
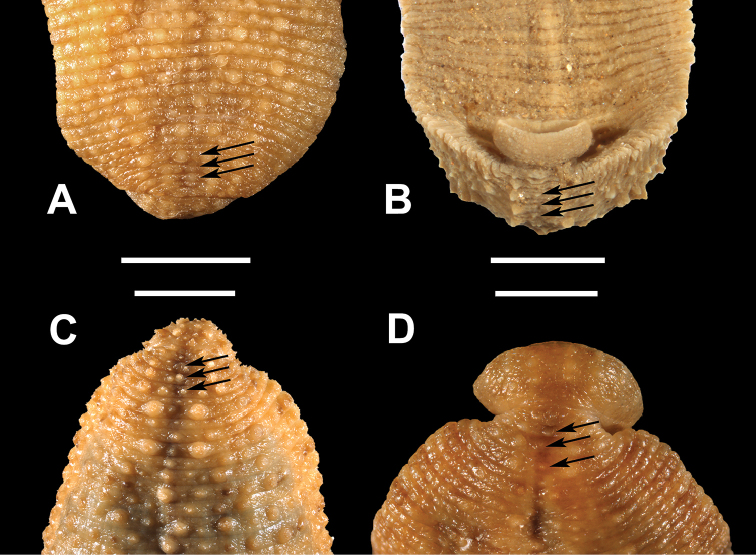
Dorsal surface, anal region. Medial row of small but distinct papillae (indicated by arrows) lying between the anus and commencement of prominent paramedial papillae, on **A** *Placobdella rugosa* (YPM 083787) **B** *Placobdella ornata*, syntype (YPM 000256) **C** *Placobdella ali* (YPM 058254) **D** *Placobdella multilineata* (YPM 083782). Scale bars: 3 mm (**A**), 1 mm (**B**), 4 mm (**C**), 2 mm (**D**).

**Figure 5.**
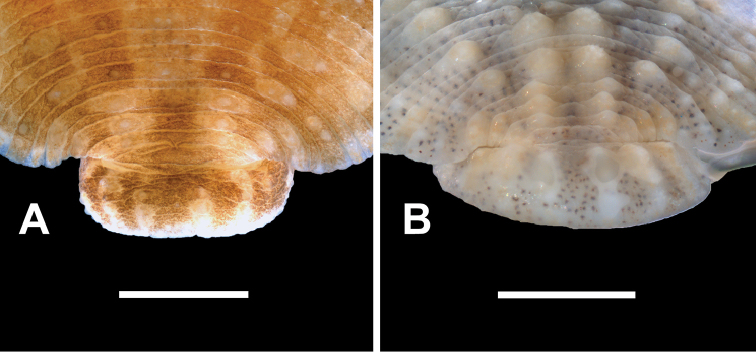
Dorsal surface, anal region, (note lack of papillae between anus and commencement of prominent paramedial papillae) of **A** *Placobdella papillifera* (YPM 083792) **B** *Placobdella siddalli* Richardson & Moser (YPM 083857). Scale bars: 1 mm.

**Figure 6.**
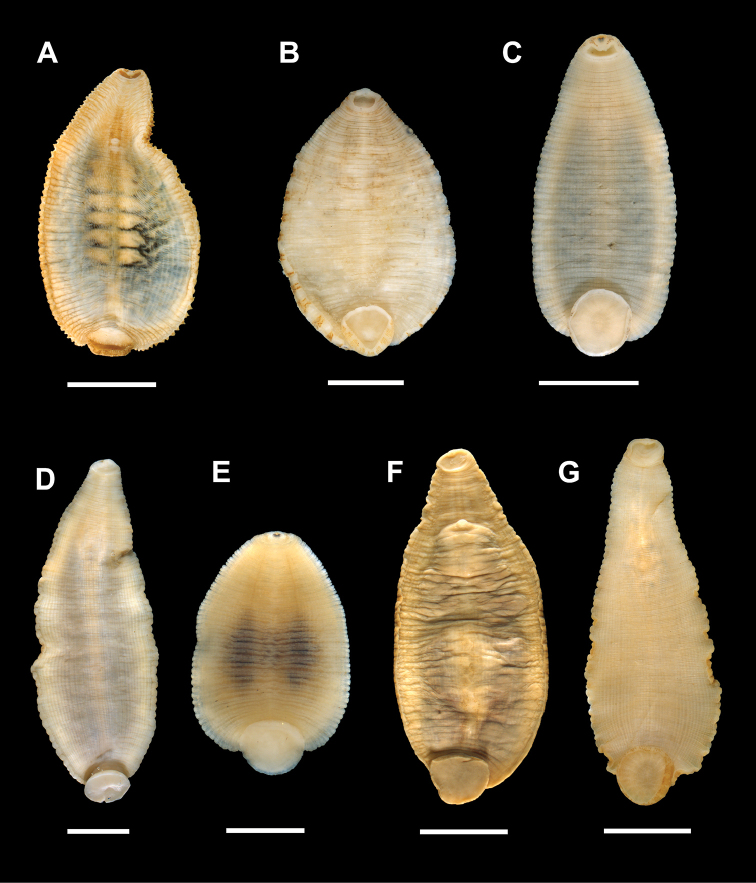
Ventral surface of various species of *Placobdella*. Note the diameter of the caudal sucker relative to body length and width of individuals. **A** *Placobdella ali* (YPM 058279) **B** *Placobdella ornata* (YPM 083847) **C** *Placobdella siddalli* Richardson & Moser (YPM 083857) **D** *Placobdella multilineata* (YPM 083850) **E** *Placobdella papillifera* (YPM 083856) **F** *Placobdella parasitica* (YPM 059092) **G** *Placobdella rugosa* (YPM 056680). Scale bars: 10 mm (**A, F**), 2 mm (**B**), 3 mm (**C**), 5 mm (**D, G**), 1 mm (**E**).

## Discussion

*Placobdella siddalli* Richardson & Moser most closely resembles *P. multilineata*, *P. ali*, and *P. rugosa*. Both *P. ali* and *P. rugosa* have faint but distinct brown pigmented lines corresponding to paralateral and paramedial papillae, that are lacking in *P. siddalli* Richardson & Moser. In *P. ali*, the dorso-medial line is unbroken, whereas it is broken in *P. siddalli* Richardson & Moser. Also *P. ali*, *P. multilineata*, *P. ornata*, and *P. rugosa*, have a medial row of small but distinct papillae, each lying between the anus and four prominent paramedial papillae (Fig. 4). Probably because of their diminutive size, these papillae have not previously been described although they are evident in Fig. 2 of Moser, Richardson, Hammond, and Lazo-Wasem (2012) and Fig. 3 of Moser, Richardson, Hammond, Govedich, and Lazo-Wasem (2012). These papillae are lacking in *P. siddalli* Richardson & Moser and *P. papillifera* (Fig. 5). *Placobdella ali* also exhibits ventral striping that is lacking in *P. siddalli* Richardson & Moser.

The relative diameter of the caudal sucker in comparison to body width and body length was found to be helpful in differentiating species of the genus *Placobdella* (Fig. 6). Table 1 gives relative size of the caudal suckers in comparison to body width and length for *P. ali*, *P. siddalli* Richardson & Moser, *P. multilineata*, *P. ornata*, *P. papillifera*, *P. parasitica*, and *P. rugosa*. The caudal sucker diameter of *P. siddalli* Richardson & Moser is 18% to 23% of the length of the body. This relative diameter is similar to that of *P. rugosa* and *P. papillifera*, but is greater than that of *P. ali* and *P. multilineata*, with the caudal sucker diameter to body-length ratio not overlapping. Likewise the diameter to body-length ratio of the caudal sucker of *P. siddalli* Richardson & Moser is larger than that of *P. ornata* and *P. parasitica*, overlapping only slightly. The caudal sucker diameter of *P. siddalli* Richardson & Moser is 40% to 54% of the width of the body. This relative diameter is greater than that of *P. ali* and *P. ornata*, with the caudal sucker diameter to body-width ratio not overlapping.

The unique color patterning, papillation and large relative size of the caudal sucker renders *P. siddalli* Richardson & Moser readily discernible from all described species in the genus *Placobdella*. It is likely that further collection, and retrospective examination of museum holdings, of the papillated *Placobdella* will provide additional information on the distribution and host utilization patterns of this intriguing new species.

In the course of this study, two new taxonomic characters have been utilized for differentiation of species within the genus *Placobdella*: the presence or absence of a medial row of small but distinct papillae lying between the anus and 4 prominent paramedial papillae and the ratio of sucker diameter to body length and width. These characters may help provide resolution between other species in the genus *Placobdella*, as well as species representing other genera.

**Table 1.** Ratio of diameter of caudal sucker to body length and width for seven species in the genus *Placobdella*.

Species	Caudal sucker diameter/Body length	Caudal sucker diameter/Maximum body width
*P. ali*	0.12–0.17	0.27–0.33
*P. siddalli*	0.18–0.23	0.40–0.54
*P. multilineata*	0.11–0.15	0.32–0.47
*P. ornata*	0.13–0.19	0.23–0.38
*P. papillifera*	0.12–0.24	0.27–0.44
*P. parasitica*	0.11–0.19	0.32–0.49
*P. rugosa*	0.07–0.24	0.31–0.47

## Supplementary Material

XML Treatment for Placobdella siddalli
